# Susceptibility of Conventional and Organic Chicken Breast and Thigh Meat to Lipid and Protein Oxidation During Heating and In Vitro Digestion

**DOI:** 10.3390/foods14193375

**Published:** 2025-09-29

**Authors:** Zeshan Ali, Thomas Van Hecke, Els Vossen, Massimiliano Petracci, Cécile Berri, Eline Kowalski, Stefaan De Smet

**Affiliations:** 1 Laboratory for Animal Nutrition and Animal Product Quality, Department of Animal Sciences and Aquatic Ecology, Ghent University, Coupure Links 653, 9000 Ghent, Belgium; zeshan.ali@ugent.be (Z.A.); thomas.vanhecke@ugent.be (T.V.H.); els.vossen@ugent.be (E.V.); eline.kowalski@ugent.be (E.K.); 2 Department of Agricultural and Food Sciences, University of Bologna, 47522 Cesena, Italy; m.petracci@unibo.it; 3 INRAE, Université de Tours, BOA, 37380 Nouzilly, France; cecile.berri@inrae.fr

**Keywords:** 4-hydroxy-2-nonenal, protein carbonyl compounds, wooden breast, antioxidants, PUFA, gastrointestinal digestion

## Abstract

It was hypothesized that differences in production system and muscle type may influence the formation of lipid oxidation products (LOP) as well as protein oxidation (protein carbonyl compounds, PCC) during the in vitro gastrointestinal digestion of chicken meat. To test our hypothesis, we investigated the formation of LOP and PCC after heating and in vitro gastrointestinal digestion of conventional and organic chicken breast and thigh meat and Wooden Breast meat. Prior to the in vitro digestion, thigh and breast meat was minced and heated. Digests of organic thigh meat had significantly higher levels of all LOP measured compared to conventional thigh meat (between +37% and +173%). Lower levels of LOP were found in digests of breast meat regardless of the production system and Wooden Breast phenotype. LOP correlated positively with heme-Fe and polyunsaturated fatty acids, negatively with anserine, and not with carnosine and α-tocopherol. PCC levels were significantly higher in thigh meat than in breast meat after heating (+43%) and digestion (+25%), irrespective of the production system. Overall, organic thigh meat exhibited the highest oxidative sensitivity during digestion. The cut-dependent differences in composition and oxidative susceptibility between organic and conventional chicken highlight the need for further research to assess potential health implications.

## 1. Introduction

Poultry farming has witnessed remarkable growth over the past two decades and remains one of the fastest-growing livestock industries worldwide. In 2024, global chicken meat production reached approximately 146 million tonnes, marking a 0.8% increase from the previous year [1]. Worldwide, to meet this high and rising demand, the majority of chicken meat (e.g., 90% in European Union countries) is produced in intensive production systems characterized by fast-growing chickens and indoor housing [2,3]. However, the success of the selective breeding programs in attaining fast growth and large breast mass has coincided with an increased occurrence of muscle myopathies [4]. Among these, the Wooden Breast myopathy has gained attention as a muscular defect characterized by hardened ridges, a paler color, and surface hemorrhagic lesions on the Pectoralis major muscle [5]. Although the Wooden Breast myopathy is common in intensive chicken production systems, its reported prevalence varies widely between countries, studies, and severity levels. In recent studies, severe Wooden Breast prevalence has been reported to range from approximately 7% to 12%, which can lead to reduced consumer acceptance and product depreciation [4,6]. On the other hand, consumer interest in alternative production systems with slower-growing birds and outdoor access (e.g., organic production) has increased in recent years, based on the perception of improved welfare compared to conventional, intensive production systems [7,8].

These contrasting production systems differ in genetics, feeding, and housing practices, which can significantly influence meat quality and nutritional value [9]. For example, meat from slower-growing chickens whether raised in organic systems or not, generally contains higher proportions of polyunsaturated fatty acids (PUFA) and higher contents of heme-iron (heme-Fe), protein, α-tocopherol, and histidine-containing dipeptides (HCD) such as carnosine and anserine, compared to conventionally produced chicken meat [7,10,11,12]. Nutritional differences also exist between chicken meat, with thigh meat containing more fat and heme-Fe and a higher proportion of PUFA than breast meat [9]. Additionally, Wooden Breast meat has a lower protein content and higher lipid content than Normal Breast meat [13].

Such variations in meat composition may have direct implications for the oxidative stability of meat, not only during storage and heating, but also during gastrointestinal digestion. For instance, PUFA are essential nutrients for human health; however, when present in combination with heme-Fe in meat, their susceptibility to oxidative reactions leads to the formation of potentially harmful lipid and protein oxidation products [14,15]. Oxidation of n-6 PUFA produces 4-hydroxy-2-nonenal (4-HNE) and hexanal (HEX), whereas oxidation of n-3 PUFA generates propanal (PROP). Both types of PUFA can generate malondialdehyde (MDA). MDA and 4-HNE are highly reactive compounds. The alkanals HEX and PROP are less reactive and have more limited effects in the body but are widely used to assess the extent of lipid oxidation. These lipid oxidation products (LOP) are more readily formed during the digestion of meat containing PUFA in the presence of heme-Fe. The pro-oxidant effects of heme-Fe were shown during in vitro digestion of chicken, pork and beef minced with lard to standardize the fatty acid (FA) profile [16]. Steppeler et al. [17] reported that higher levels of MDA and n-3 PUFA-derived oxidation products are formed during the digestion of fish and chicken meat compared to beef, due to their higher levels of PUFA. Furthermore, the interaction of amino acids with reactive oxygen species (ROS), LOP or Maillard reaction products results in the formation of protein carbonyl compounds (PCC), which are established markers of oxidative damage to proteins [18].

Excessive formation and intake of oxidation products may overwhelm the body’s antioxidant defense system, potentially causing oxidative stress and human health issues. For example, dark meat consumption was linked to increased postprandial plasma MDA and oxidized low density lipoproteins (LDL) levels in healthy volunteers, which may be associated with cardiovascular diseases [19]. However, this largely depends on the concentration of LOP. At lower concentrations in biological tissues or plasma (0.1–1 µmol/L), 4-HNE may exhibit hormetic effects. In contrast, moderate (1–10 µmol/L) and higher (up to 20 µmol/L) concentrations may lead to pathophysiological effects [20]. Similarly, recent studies suggest a role of dietary protein oxidation in aging and age-related diseases in humans [21]. This concern is particularly relevant given the growing consumer preference for organic and alternative production systems, which are often perceived as healthier options. Although previous studies have compared oxidative stability during storage across systems, there remains a knowledge gap on oxidation during gastrointestinal digestion of chicken meat produced in various production systems. The altered meat composition of the Wooden Breast myopathy may also affect its oxidative stability. Therefore, this study was designed to address this gap by assessing the oxidative stability during heating and in vitro gastrointestinal digestion of various chicken meats.

The current study comprised two experiments. We compared the oxidative stability of meat from two production systems (conventional vs. organic) and meat types (breast vs. thigh) in Experiment 1, and Wooden Breast and Normal Breast phenotypes within the conventional system in Experiment 2. In both experiments, we measured meat composition variables (heme-Fe, PUFA, α-tocopherol, carnosine, and anserine), LOP (4-HNE, HEX, PROP, TBARS), and PCC in heated meats and their corresponding in vitro gastrointestinal digests.

## 2. Materials and Methods

### 2.1. Chemicals

All digestive enzymes (α-amylase from hog pancreas [~50 U/mg; 10080], mucin from porcine stomach type II [M2378], pepsin from porcine gastric mucosa [>250 U/mg solid, P7000], lipase from porcine pancreas type II [10–400 U/mg protein; L3126], pancreatin from porcine pancreas [8 × United States Pharmacopeia specifications; P7545], and porcine bile extract [B8631]), the reagents (2-thiobarbituric acid [T5500], 1,3-cyclohexanedione [C101605], and 2,4-dinitrophenylhydrazine [D199303], and the analytical standards (HNE-DMA [purity > 85%; H9538], 1,1,3,3-Tetramethoxypropane [purity > 99%; 108383], alpha-tocopherol [purity > 99%; PHR1031], hexanal [purity ≥ 98%; 115606] and propanal [purity ≥ 98%; 64409]) were purchased from Merck in Diegem, Belgium. The analytical standards of L-carnosine and L-anserine were chemically synthesized by Flamma S.p.A. (Chignolo d’Isola, Bergamo, Italy). All other common chemicals were purchased from AnalytiChem Belgium NV (Zedelgem, Belgium).

### 2.2. Meat Sampling and Experimental Design

In Experiment 1 of the present study, meat from two distinct broiler production systems was used, i.e., conventional intensive (CON) and organic farming (ORG). The differences between these systems and the finisher phase diets are detailed in Table 1. Housing conditions in the CON system adhered to Council Directive 2007/43/EC [22], with temperature, humidity, and light conditions meticulously controlled, and a stocking density typically ranging from 33 kg/m^2^ up to 42 kg/m^2^. In contrast, chickens in the ORG system were housed and reared according to the EU Commission Regulation 889/2008 [23], which mandates access to fresh air, daylight, and outdoor space, with a minimum of 4 m^2^ of outdoor run per bird. Due to an avian influenza outbreak in Belgium during the sampling period, outdoor access was temporarily restricted on some ORG farms. This reduced the planned 6-week duration to 5, 4, 3, 2, and 1 weeks in the five farms. Chickens in both production systems were fed ad libitum. Five farms, distributed across Belgium, were selected from each production system. A total of 10 chickens per production system (two from each farm, randomly selected without regard to sex) were sampled after slaughtering in two slaughterhouses. The carcasses were transported to the laboratory in a cooling truck. On the second day post-slaughter, approximately 100 g of meat from the breast (skinless Pectoralis major) and thigh cuts were sampled for analyses and in vitro gastrointestinal digestion. Carcasses with signs of Wooden Breast were excluded.

In Experiment 2, chickens displaying Wooden Breast were selected from the same five CON farms of Experiment 1 (*n* = 10, two per farm). Wooden Breast was identified following the protocol of Tijare et al. [24] on a 4-point scale (0 = normal, 1 = mild, 2 = moderate, 3 = severe). To allow for more precise differentiation, a score of 2.5 was included to represent samples of which the severity condition was greater than moderate but not severe. Only carcasses with at least a score of 2 were selected, including 7 samples with a score of 2.5, 2 with a score of 3, and 1 with a score of 2. The breast was incised from the most affected part (tip of the breast). These Wooden Breast meat samples were compared to the similarly sampled tip of the Normal Breast samples (*n* = 10) of the CON system from Experiment 1.

All meat samples were vacuum-packed and stored frozen at −80 °C until digestion and analysis. Samples were collectively defrosted and minced. Before the in vitro gastrointestinal digestion, 100 g of each sample was finely minced (Moulinex DP810; Moulinex, Paris, France) and heated at an oven temperature of 180 °C (Memmert UN110; Memmert, Schwabach, Germany) until the core temperature reached 70 °C. Following the heating procedure, meats were minced again, vacuum packed, and stored at −80 °C until digestion or analysis. The characterization of meat was performed on heated meat samples, whereas the analyses of lipid oxidation (TBARS, 4-HNE, HEX, PROP) and protein oxidation products (PCC) were conducted on both the heated meats and their corresponding digests.

### 2.3. In Vitro Digestion

The in vitro gastrointestinal digestion was based on the protocol of Versantvoort et al. [25], as adapted for studying oxidative reactions in the gastrointestinal system by Van Hecke et al. [26]. The composition and concentration of digestive juices are shown in Supplementary data (Table S1). This static in vitro model simulated digestion in the mouth, stomach, and small intestine aerobically at 37 °C. Briefly, 4.5 g of meat underwent simulated digestion, starting with a 5 min exposure to 6 mL of saliva, followed by 2 h in 12 mL of gastric fluid, and concluding with 2 h incubation following the consecutive addition of 2 mL bicarbonate buffer, 12 mL duodenal fluid, and 6 mL bile. In the incubator, bottles were protected from light. After digestion, all samples were homogenized at 9500 rpm for 5 s, and the resulting aliquots (termed digests) were stored at −80 °C for further analysis. All samples were digested on the same day with the same digestive juices to exclude possible day effects.

### 2.4. Meat Composition Analyses

All analyses of meat composition were performed on heated meat samples.

#### 2.4.1. Fatty Acids

Lipids were extracted in duplicate from 3 g of minced meat, using chloroform/methanol (2:1, *v*/*v*) following a modified method of Folch et al. [27], after which FA were analyzed as described by Raes et al. [28]. After methylation with NaOH/MeOH followed by HCl/MeOH, fatty acid methyl esters (FAME) were analyzed using a gas chromatograph (HP 7890A, Agilent Technologies, Diegem, Belgium) equipped with a fused silica SP-2560 capillary column (75 m × 0.18 mm i.d. × 0.14 µm thickness; Supelco Analytical, Bellefonte, PA, USA) and a flame ionization detector. Nonadecanoic acid (C19:0) was added as an internal standard to quantify the FA.

#### 2.4.2. Heme-Fe

Heme-Fe was quantified by measuring the hematin concentration in duplicate as described by Hornsey [29]. In brief, 5 g of minced meat was weighed into a 100 mL plastic bottle covered with aluminum foil to protect from light. Hematin was then extracted using acetone, water, and HCl (40:2:1). The extract was left in a dark place at room temperature for 1 h to allow all heme pigments to be converted to acid hematin. After this, the extract was filtered through filter paper (S&S 589^2^) and absorbance was measured spectrophotometrically at 640 nm (Genesys 10S UV–VIS, Thermo Scientific, Madison, WI, USA). Hematin content was converted to heme-Fe using the formula: heme-Fe = (hematin concentration × atomic weight of Fe)/molecular weight of hematin.

#### 2.4.3. α-Tocopherol

The analysis of α-tocopherol was conducted according to Claeys et al. [30]. In brief, 2 g of meat (in duplicate) was placed in brown tubes to protect from light and homogenized in ethanol at 78 °C for 30 min. The cooled extract was centrifuged with KCl, extracted twice with hexane, and evaporated under nitrogen. The resulting fraction and standards (0–20 μg/mL) were injected in brown vials, into an HPLC system (Agilent 1200 series, Diegem, Belgium) equipped with a Supelcosil LC18 column (25 cm × 4.6 mm, 5 μm) and a UV detector. The absorbance was measured at 292 nm, and α-tocopherol content was calculated by comparing peak areas with a standard curve.

#### 2.4.4. Carnosine and Anserine

Carnosine and anserine concentrations were determined using a modified method of Kobe et al. [31]. Briefly, 1 g of minced meat in duplicate was homogenized in 45 mL of 0.01 mol/L phosphate buffer (pH 7.4) with an ultra-turrax (Type 18/10, Janke and Kunkel) at 9500 rpm for 30 s. After adding 1 mL of acetonitrile to 2 mL of the homogenate, the mixture was stored overnight at 4 °C and centrifuged at 3000 rpm for 10 min at 4 °C. The supernatant was filtered and 20 μL was injected into an HPLC column (EC250/4.6 Nucleosil 120-7 NH2, Machery-Nagel, Duren, Germany). Concentrations were determined by comparing peaks to standard solutions.

### 2.5. Oxidation Products in Meats and Digests

#### 2.5.1. Lipid Oxidation Products

Concentrations of 4-HNE, HEX, and PROP in meat and digest samples were measured as fluorescent derivatives using 1,3-cyclohexanedione (CHD) through HPLC (Agilent 1200 series, Diegem, Belgium), following a method modified by Van Hecke et al. [32]. In pre-heated glass tubes, digests and meat samples were mixed with MeOH and CHD reagent, vortexed, and incubated in duplicate. After cooling, MeOH was added, and the mixture was filtered into dark HPLC vials. Separation was performed using a Supelcosil LC-18 column (25 cm × 4.6 mm, 5 μm, catalog no. 58295, Supelcosil), and aldehydes were detected with a fluorescence detector.

Thiobarbituric acid reactive substances (TBARS) in meats and digests were measured as MDA-equivalents according to the method of Grotto et al. [33]. Following alkaline hydrolysis, the absorbance of the MDA-TBA complex was measured spectrophotometrically at 532 nm in the n-butanol fraction (Genesys 10S UV–VIS, Thermo Scientific, Madison, WI, USA).

#### 2.5.2. Protein Oxidation Products

Protein oxidation in meat and digest samples was assessed by measuring PCC following the method of Ganhão et al. [34]. Three aliquots of digest samples (700 μL) or homogenized meat (200 μL from the 1:10 meat-to-phosphate-buffered solution) were treated with trichloroacetic acid to precipitate proteins. One aliquot was used as a blank, while the others were treated with 2,4-dinitrophenylhydrazine (DNPH) to form stable 2,4-dinitrophenylhydrazone complexes. After incubation (1 h shaking protected from light), excess DNPH and fat were removed by three washes with ethanol/ethyl acetate. Afterwards, guanidine HCL (6 M) was added to dissolve proteins. Absorbance was measured spectrophotometrically at 280 nm and 370 nm (Genesys 10S UV–VIS, Thermo Scientific, Madison, USA). PCC concentrations were calculated by measuring the amount of 2,4-DNPH incorporated per mg of protein, using the following formula:

C_hydrazone_/C_protein_ = A_370_/(ε_hydrazone370_ × (A_280_ − A_370_ × 0.43)) × 1000

### 2.6. Statistical Analysis

All analyses were conducted using R (version 4.3.1) and RStudio (version 2023.09.0). Data visualization was performed using the ggplot2 package. In Experiment 1, data were analyzed using a two-way analysis of variance (ANOVA) model, which evaluated the main effects of production system (CON vs. ORG) and meat type (breast vs. thigh), as well as their interaction term. The ANOVA model was fitted using the ‘aov’ function in R. Residual normality was assessed through Q-Q plots, histograms, and the Shapiro–Wilk test, and data was log-transformed when needed. Estimated marginal means were calculated using the emmeans package, and Bonferroni-adjusted post hoc tests were performed on all pairwise comparisons. A heatmap of Pearson correlation coefficients was generated using the pheatmap package. Principal Component Analysis (PCA) was conducted on the standardized numeric variables from the dataset using the ‘prcomp’ function. A biplot was generated to illustrate the PCA results, highlighting individual observations along with their respective groups. The significance level was set at *p* ≤ 0.05. A *p*-value greater than 0.05 but less than or equal to 0.1 was considered a trend.

In Experiment 2, a two-sample *t*-test was applied to assess mean differences between the two meat types (Normal Breast and Wooden Breast). The Shapiro–Wilk test for normality and Levene’s test for homogeneity of variances confirmed the assumptions for the *t*-test. The significance level was set at *p* ≤ 0.05.

## 3. Results

### 3.1. Experiment 1: Meat Composition

**Figure 1:**
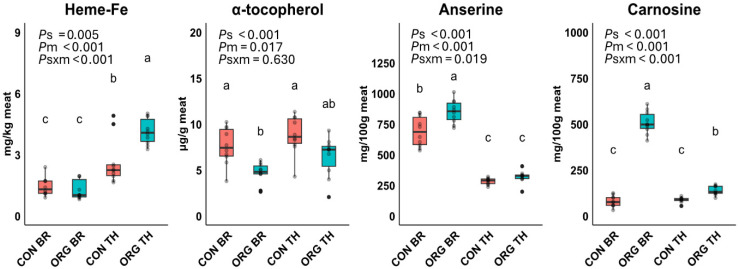
Effect of production system (s), meat type (m), and their interaction term (s × m) on heme-Fe, α-tocopherol, carnosine and anserine in conventional breast (CON BR), organic breast (ORG BR), conventional thigh (CON TH) and organic thigh (ORG TH) in Experiment 1 (*n* = 10 each). Means with different superscripts are significantly different. *p*-values for the main effects of production system (*P*s), meat type (*P*m), and their interaction (*P*s × m) were derived from an ANOVA model.

Table 2 summarizes the fatty acid profile, while Figure 1 shows concentrations of heme-Fe, α-tocopherol, carnosine, and anserine. The concentrations of total FA, SFA and MUFA were significantly influenced by the meat type only, being approximately three times higher in thigh meat compared to breast meat from both production systems. Levels of PUFA, encompassing both n-3 and n-6 PUFA, were mainly affected by the meat type, but significant effects of the production system, alongside its interaction with meat type, were observed as well. The lowest concentrations of total n-3 and total n-6 PUFA were observed in breast meat from both production systems followed by intermediate levels in CON thigh and the highest levels in ORG thigh. This pattern of n-3 PUFA and n-6 PUFA was reflected in the pattern of the levels of α-linolenic acid (ALA) and linoleic acid (LA) as the parent fatty acids, respectively. The long-chain n-3 derivative EPA was significantly higher in the CON thigh compared to the ORG breast with intermediate levels for the other groups. The level of DHA significantly increased in the order CON breast and CON thigh < ORG breast < ORG thigh. The n-6/n-3 PUFA ratio was not different between the groups.

The concentration of heme-Fe in the meats was significantly influenced by the production system, meat type and their interaction. The levels of heme-Fe were highest in ORG thighs, followed by CON thighs, and lowest in the breast meat irrespective of production system. Levels of α-tocopherol were significantly lower in breast compared to thigh meat (−26%), and higher in meats from the CON system compared to ORG meats (+48%). Anserine and carnosine concentrations were significantly affected by the production system, meat type, and their interaction. Specifically, breast meat had significantly higher levels of anserine (+157%) and carnosine (+161%) than thigh meat. ORG meats contained higher levels of anserine (+20%) and carnosine (+287%) than CON meats.

### 3.2. Experiment 1: Lipid and Protein Oxidation Products

**Figure 2:**
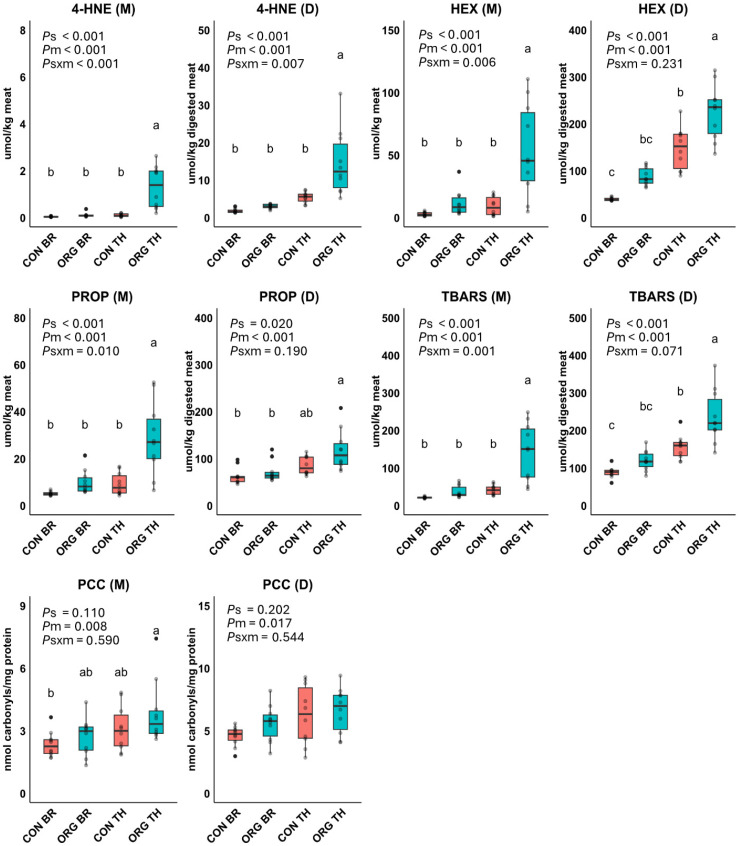
Effect of production system (s), meat type (m), and their interaction term (s × m) on products of lipid oxidation (4-hydroxy-2-nonenal, 4-HNE; hexanal, HEX; propanal, PROP; thiobarbituric acid reactive substances, TBARS) and protein oxidation (protein carbonyl compounds, PCC) in meats (M) and digests (D) of conventional breast (CON BR), organic breast (ORG BR), conventional thigh (CON TH) and organic thigh (ORG TH) in Experiment 1 (*n* = 10 each). Means with different superscripts are significantly different. *p*-values for the main effects of production system (*P*s), meat type (*P*m), and their interaction (*P*s × m) were derived from an ANOVA model.

Concentrations of 4-HNE, HEX, PROP, TBARS and PCC in meats (before digestion) and in digests (post-digestion) are presented in Figure 2. As anticipated, the levels of metabolites associated with the oxidation of n-6 PUFA (4-HNE and HEX), n-3 PUFA (PROP), and both n-6 and n-3 PUFA (TBARS) were multi-fold increased during the in vitro gastrointestinal digestion process. Levels of LOP in meats and digests were significantly affected by meat type and production system, with significant interaction effects observed primarily in the meats. ORG thigh contained significantly higher levels of all analyzed LOP, with increases ranging from 3.5 to 18 times in the heated meat samples and 1.6 to 4.4 times in the digests, compared to the other meat types which did not differ among each other. Specifically, the digests of ORG thigh contained significantly higher concentrations of 4-HNE (4.4-fold), HEX (2.4-fold), PROP (1.6-fold) and TBARS (2-fold) compared to the mean of other digests. Chicken thigh meat from the ORG system had significantly higher PCC levels compared to CON breast, whereas intermediate levels were found in the ORG breast and CON thigh meat. After digestion, PCC increased significantly, demonstrating a meat type effect, with digests of thigh exhibiting a higher (+25%) concentration of PCC compared to digests of breast.

### 3.3. Experiment 1: Relationships Among Compositional Variables and Oxidation Products

**Figure 3:**
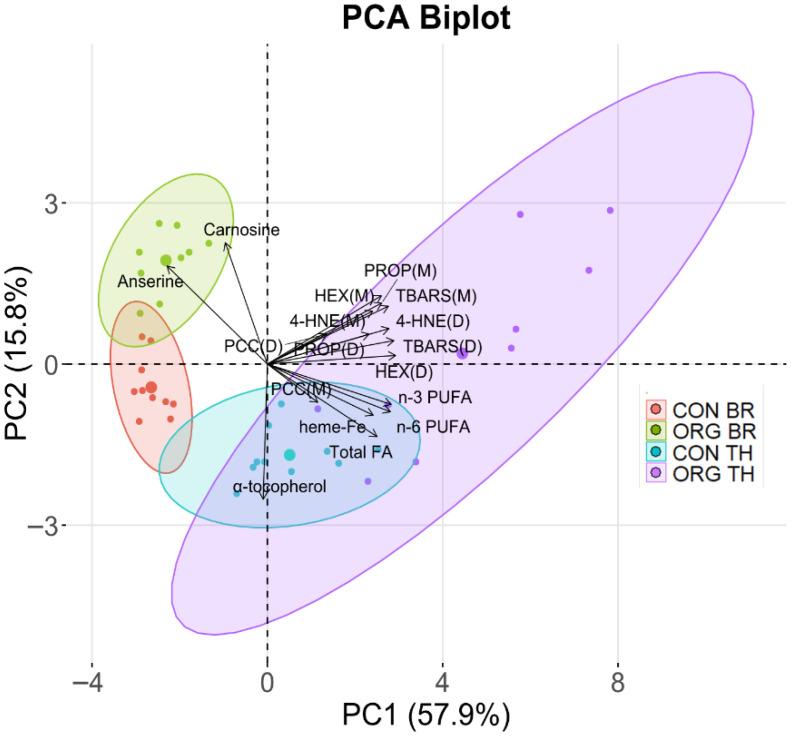
Principal component analysis (PCA) biplot from Experiment 1, showing the distribution of oxidation products (4-hydroxy-2-nonenal, 4-HNE; hexanal, HEX; propanal, PROP; thiobarbituric acid reactive substances, TBARS; protein carbonyl compounds, PCC) in meats (M) and digests (D), as well as meat composition variables (n-6 PUFA, n-3 PUFA, total FA, heme-Fe, α-tocopherol, anserine, carnosine) across experimental groups: conventional breast (CON BR), conventional thigh (CON TH), organic breast (ORG BR) and organic thigh (ORG TH). The ellipse around each group represents the 95% confidence interval, highlighting the separation between groups. The percentage of variance explained by each principal component (PC1 and PC2) is indicated in parentheses.

Pearson correlation coefficients were used to assess relationships among the variables across all meats or digests, as shown in Supplementary data (Figure S1). 4-HNE, HEX, PROP and TBARS in meats and digests were positively correlated with heme-Fe, total FA, n-3 PUFA, and n-6 PUFA. In digests, PCC were positively correlated with 4-HNE, HEX, PROP and TBARS. The antioxidants (α-tocopherol, carnosine and anserine) showed generally negative correlations with oxidation products (4-HNE, HEX, PROP, TBARS, and PCC) in meats and digests. To further investigate these relationships, a PCA biplot was performed as shown in Figure 3. A total of 73.7% of the variance in the data was explained, with PC1 accounting for 57.9% and PC2 for 15.8%. PC1 primarily separated the groups based on LOP and FA profile. Markers of lipid oxidation loaded heavily along this dimension and were positively associated with n-3 PUFA, n-6 PUFA, and heme-Fe. In contrast, PC2 distinguished groups based on antioxidant content. The positioning of the groups along these components reflected differences in nutrient composition and oxidative profiles, with a more distinct separation between meat types than between production systems.

### 3.4. Experiment 2: Meat Composition

**Figure 4:**
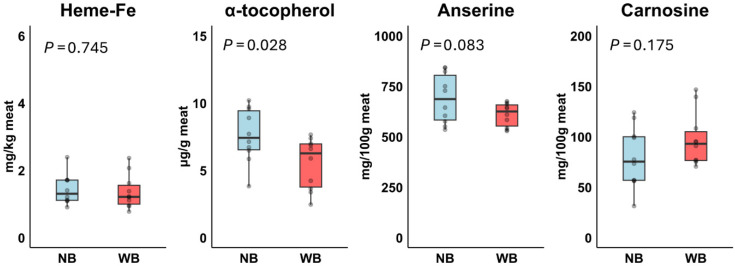
Effect of Normal Breast (NB; *n* = 10) and Wooden Breast (WB; *n* = 10) phenotype on heme-Fe, α-tocopherol, carnosine and anserine in meats in Experiment 2. *p*-values were based on *t*-test.

No significant differences were observed for total FA, the n-6/n-3 PUFA ratio, and the various FA fractions between Normal Breast and Wooden Breast, except for ALA, which was significantly higher in Wooden Breast (Table 3). This higher ALA contributed to a tendency toward a +45% increase in n-3 PUFA content in Wooden Breast meat. In addition, Wooden Breast meat contained significantly lower α-tocopherol levels (−28%) and tended to have lower anserine levels compared to Normal Breast meat, whereas no significant differences were observed for heme-Fe and carnosine (Figure 4).

### 3.5. Experiment 2: Lipid and Protein Oxidation Products

**Figure 5:**
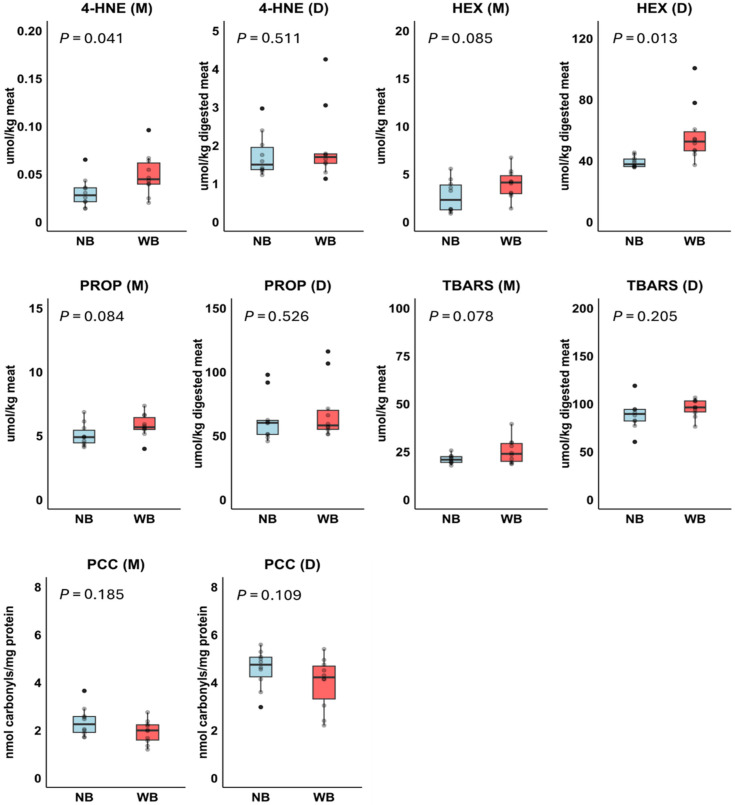
Effect of Normal Breast (NB; *n* = 10) and Wooden Breast (WB; *n* = 10) phenotype on products of lipid oxidation (4-hydroxy-2-nonenal, 4-HNE; hexanal, HEX; propanal, PROP, thiobarbituric acid reactive substances, TBARS) and protein oxidation (protein carbonyl compounds, PCC) in meats (M) and digests (D) in Experiment 2. *p*-values were based on *t*-test.

Concentrations of 4-HNE, HEX, PROP, TBARS and PCC in meats and digests are presented in Figure 5. In the meats, concentrations of 4-HNE, TBARS, PROP, and HEX were slightly higher in Wooden Breast compared to Normal Breast, with statistically significant differences for 4-HNE and trends observed for TBARS, PROP, and HEX. After digestion, the concentration of these oxidation products was generally increased. However, only HEX levels were significantly higher in Wooden Breast compared to Normal Breast.

## 4. Discussion

We investigated oxidative reactions in chicken meat during heating and in vitro gastrointestinal digestion, focusing on breast and thigh meat from CON and ORG production systems, as well as Wooden Breast and Normal Breast meat within a CON system. One of the major outcomes was the higher formation of LOP in thigh meat from ORG chickens during in vitro digestion. This appears to be largely driven by variations in meat composition which were influenced by both the production system and meat type. Although ORG meat may have a more favorable nutritional profile due to the rearing and feeding conditions, its higher susceptibility to oxidation may challenge the widespread belief that it is inherently superior for human health. However, one should be cautious in generalizing our findings to all ORG chicken meat and in attributing the differences to a specific factor (e.g., breed) because of the multiple interrelated factors characterizing production systems, as will be discussed below. Nevertheless, comparing chicken meat from different commercial systems has scientific and practical relevance as these meats reflect field conditions under which consumers purchase meat.

Higher proportions of PUFA (n-3 and n-6 PUFA) and heme-Fe content have been observed in ORG chicken meat [7,10,11], which aligns with the results of the present study. Furthermore, thigh meat, compared to breast meat, has been reported to contain higher levels of PUFA and heme-Fe [9]. The higher heme-Fe content in thigh meat is associated with a greater proportion of oxidative muscle fibers compared to the predominant glycolytic fiber type in breast muscle [35]. In the present study, the combined effect of production system and meat type pronounced the differences in concentration of heme-Fe. Heme-Fe was reported to be higher in slow-growing chickens, particularly in thigh muscles compared to breast muscles [36].

In contrast to studies that reported elevated α-tocopherol levels in meat from ORG chickens due to increased foraging behavior [37], we observed lower α-tocopherol concentrations in ORG meat, consistent with the findings of Michiels et al. [38]. Since the feed α-tocopherol content was similar, reduced levels may reflect greater utilization as an antioxidant to counteract elevated oxidative stress from increased physical activity [39,40]. Supporting this, exploratory outdoor chickens exhibited lower plasma tocopherol levels and total antioxidant status than sedentary counterparts [41]. In fact, oxidative homeostasis depends on the dietary intake of antioxidants, but also on the interaction of the tissue lipid profile with oxidative stress produced by kinetic activity [42]. The latter is related to the effect of locomotory activity on muscle metabolism and the production of free radicals. However, metabolism and adaptation to exercise can be affected by genetic strain, which in turn may alter the body antioxidant status [43,44]. In addition, the limited outdoor access of ORG chickens in this study could have restricted their foraging behavior, thereby also reducing their intake of natural antioxidants. The concentration of anserine in the heated meats was notably higher than that of carnosine, confirming that anserine is the most abundant HCD in chicken skeletal muscle [12]. The higher concentration of HCD in breast meat compared to thigh, particularly in ORG breast, is likely related to the predominance of type IIB fast-twitch glycolytic fibers. These fibers rely on anaerobic metabolism, leading to increased lactic acid production, which necessitates more buffering compounds like anserine and carnosine to maintain the pH balance [45].

We observed considerable within-group variability for oxidation products. This likely reflects the fact that samples were obtained from multiple commercial farms. Although the farms were broadly comparable within each production system, subtle differences in factors such as outdoor access, diet composition, animal genetics, farm location, management practices, and handling of the birds may have contributed to this variability. The higher oxidative susceptibility of ORG meat after heating was likely primarily due to its higher degree of unsaturation. In line with this, Castellini et al. [39] reported higher TBARS levels in meat from ORG chickens compared to CON chickens. The meat type also plays a crucial role in oxidative stability. Thigh meat is naturally more prone to oxidation than breast meat, irrespective of the production system, and compared to other meats like beef and pork [14]. PCA in the present study confirmed these findings, with oxidation products clustering strongly with ORG thigh meat. The antioxidant profile of the meat further influenced LOP formation. Lower levels of α-tocopherol and HCD in meat were associated with higher oxidation. This is consistent with findings from Michiels et al. [38], who reported elevated lipid oxidation and reduced α-tocopherol levels in chickens raised under conditions very similar to the systems in the present study. α-tocopherol is a well-known, potent lipid-soluble antioxidant [46]. HCD have been reported to contribute to the antioxidant status of chicken meat [47]. Specifically, carnosine inhibits lipid oxidation not only by scavenging free radicals and chelating pro-oxidant metals but also by inducing structural changes in the pro-oxidant metmyoglobin, thereby reducing its oxidative activity [48]. Also in the present study, lower levels of LOP were observed in meats with higher concentrations of HCD; however, the correlations only reached significance for anserine with LOP.

Notably, the formation of LOP increased during in vitro digestion compared to the levels observed in the heated meats. This is consistent with previous studies on the in vitro digestion of various muscle foods, e.g., Gorelik et al. [19], Steppeler et al. [17], and Van Hecke et al. [32,49] reporting similar increases in LOP levels during digestion. This increase was observed in all groups. A key factor contributing to this rise may be the conditions in the gastric phase of the in vitro model, which includes low pH and aeration to mimic the stomach’s aerobic environment. These conditions are ideal for promoting lipid peroxidation and co-oxidation of dietary constituents [50]. After digestion, 4-HNE, HEX, PROP, and TBARS levels remained highest in the ORG thigh digests compared to the other digests, consistent with the heated meat results. However, digestion revealed a unique redistribution of HEX and TBARS: levels were lowest in CON breast, intermediate in ORG breast and CON thigh, and highest in ORG thigh. The observed variation difference in LOP between heated and digested meats suggests that the in vitro digestion process itself may influence HEX and TBARS levels. This indicates that, beyond the role of meat composition in oxidation during heating, additional factors present during digestion may further contribute to LOP formation. As meat moves through the distinct stages of the digestion process, it encounters specific conditions in each phase conducive to oxidative reactions, adding complexity to the formation of LOP as digestion progresses [15]. Post-digestion LOP levels were higher in thigh meat than in breast meat, consistent with findings from Van Hecke et al. [49]. Interestingly, the LOP in meats and digests exhibited similar correlations with n-3 PUFA and n-6 PUFA, whereas their precursors are either n-6 PUFA (precursor of 4-HNE and HEX) or n-3 PUFA (precursor of PROP). This may be attributed to the correlated pattern of n-3 and n-6 PUFA in the meats. In addition, the initial oxidative status of meat lipids also plays an important role. Primary oxidation products such as hydroperoxides can promote lipid peroxidation during digestion, leading to increased formation of secondary LOP [51]. Larsson et al. [52] demonstrated that higher initial oxidation levels in cod liver oil resulted in more TBARS during in vitro digestion, partly due to the depletion of endogenous antioxidants.

The observed lower levels of α-tocopherol in ORG meat may have reduced its capacity to inhibit LOP formation during in vitro digestion [51]. Supporting this, Kenmogne-Domguia et al. [53] reported significant losses of tocopherols during in vitro digestion of unsaturated oil emulsions, with α-tocopherol being rapidly consumed during the gastric phase. Moreover, the presence of pro-oxidant factors such as metmyoglobin further accelerates tocopherol depletion, thereby reducing the system’s ability to counteract lipid peroxidation. This indicates that while antioxidants are present, their capacity can be overwhelmed under highly oxidative conditions. The high PUFA and heme-Fe in ORG thigh meat likely created a strong oxidative environment that exceeded the protective potential of antioxidants. Similar findings have been reported for beef, suggesting that antioxidant defenses are effective under low oxidative conditions but insufficient when reactive substances are abundant [54]. Likewise, Van Hecke et al. [49] did not find the intrinsic levels of α-tocopherol, carnosine and anserine to be major determinants for the formation of oxidation products during in vitro digestion of meat from different species. On the other hand, the addition of extra antioxidants could partially mitigate oxidation in a study by Van Hecke et al. [55], who reported significantly lower levels of TBARS, HEX, and 4-HNE during in vitro digestion of high-fat beef mince supplemented with α-tocopherol.

Although there is limited information on the ability of antioxidant dipeptides like carnosine and anserine to reduce LOP during the digestion of muscle foods, both have shown the ability to inhibit LDL oxidation, especially when LDL was oxidized in vitro with copper in their presence [56]. In that study, carnosine was found to be a more effective aldehyde quencher than anserine at equal concentrations. In the present study, the absolute amounts of anserine were approximately three times higher than those of carnosine in breast and thigh meat, which may overshadow carnosine’s antioxidant effects. Li et al. [57] reported a dual role of carnosine as both an antioxidant and pro-oxidant during in vitro digestion of a burger meal model. At higher concentration, carnosine exhibited antioxidant properties, whereas at moderate concentration, it acted as a pro-oxidant. In addition, the antioxidant efficacy of carnosine may be influenced by other meal factors, such as the fat content and cooking conditions. For example, the ability of carnosine to reduce lipid oxidation during in vitro digestion was more pronounced in moderately cooked, low-fat (1.3%) pork compared to high-fat (10%) pork [58]. In our study, these factors may have similarly contributed to the lower LOP formation in breast, possibly due to its higher carnosine and lower fat content compared to thigh.

The elevated PCC in thigh meat compared to breast meat reinforces the idea that the greater susceptibility of oxidative-type muscles to lipid oxidation, compared to glycolytic-type muscles, also extends to protein oxidation. Another factor that could have contributed to the higher PCC in thigh meat compared to breast is that primary as well as secondary LOP may serve as substrates for protein oxidation [59]. Estévez [18] suggested that reactive LOP, such as 4-HNE, can promote the formation of PCC in meat. Similarly, Van Hecke et al. [49] reported that 4-HNE may further enhance PCC formation during in vitro digestion of meat, in line with the significant correlations and shared PCA clustering of PCC and LOP in our digests. Although Santé-L’houtellier et al. [60] reported that α-tocopherol exerts a protective effect against protein oxidation during the storage of lamb meat, we did not observe increased protein oxidation after heating and digestion of ORG meats, despite their lower α-tocopherol levels.

Minor differences in the FA profile between Wooden Breast and Normal Breast phenotypes were noticed in the present study, in line with Soglia et al. [61] and Thanatsang et al. [62], except for higher ALA concentrations in Wooden Breast, as well as a trend toward increased total n-3 PUFA levels. In partial agreement with Abasht et al. [63], we found no significant difference in carnosine, while anserine levels tended to be lower in Wooden Breast meat. The slightly higher levels of TBARS in Wooden Breast meat were consistent with the findings of Soglia et al. [61] and Xing et al. [64]. The former study indicated that although there were no differences in the FA profiles between Normal Breast and Wooden Breast, the Wooden Breast meat exhibited a greater susceptibility to lipid oxidation. In the present study, the modestly higher lipid oxidation observed in Wooden Breast may have been due to a combination of higher levels of n-3 PUFA and lower α-tocopherol levels. Other factors that could stimulate lipid oxidation in Wooden Breast phenotype include the continuous exposure of phospholipids to oxidative stress due to the hypoxic conditions followed by reoxygenation, which may enhance ROS formation [65]. Li et al. [66] underlined that high levels of lipid oxidation may be caused by increased metabolic waste, and poor blood and oxygen supply in the Wooden Breast phenotype. Although these processes occur in vivo, the oxidative damage encountered by the tissues could predispose the meat to increased lipid oxidation post-mortem or during digestion [67]. Moreover, Carvalho et al. [68] reported that Wooden Breast meat shows an altered redox balance, with increased abundance of proteins involved in oxidative damage repair and inflammation, as well as depletion of sulfur-containing protein components, which may compromise its antioxidant defense. Contrary to studies showing higher protein oxidation in Wooden Breast meat [61,62,64], our findings did not indicate significant differences in PCC between Normal and Wooden Breast meat, both before and after digestion. This may have been due to the similarities in muscle fiber composition, heme-Fe levels, and minor differences in secondary LOP between the two types of meat.

The higher susceptibility of ORG chicken meat to oxidation during digestion may compromise its nutritive value and reduce the bioavailability of nutrients (fatty acids and amino acids). This may seem counterintuitive as ORG foods are generally perceived as healthier and more nutritious due to their more ‘natural’ mode of production. However, there is a lack of comprehensive studies on the nutritional and health impact of consuming ORG versus CON meat [69]. In this context, the higher lipid oxidation observed in the present study does not necessarily translate to an increased health risk for humans. The health implications of oxidation products are complex and multifaceted, with their effects depending strongly on their concentrations. For instance, 4-HNE can modify LDL and contribute to foam cell formation, a factor in cardiovascular disease; however, at lower concentrations in plasma or vascular tissue, 4-HNE may induce adaptive cellular responses that enhance resistance to oxidative stress [20,70]. Our findings were derived from an in vitro model, which, although informative, does not fully replicate human physiological conditions. Therefore, caution is needed when extrapolating the present results to in vivo scenarios.

## 5. Conclusions

This study showed that both production system and meat type significantly influenced oxidative reactions in chicken meat during in vitro gastrointestinal digestion, although their effects were not entirely independent. Organic thigh meat contained more heme-Fe and PUFA compared to the other meats, coinciding with increased formation of LOP during in vitro digestion, whereas PCC formation was not affected. Our findings show the importance of considering oxidative processes occurring specifically during gastrointestinal digestion, beyond the usual post-harvest assessment of meat oxidative stability.

One should be cautious in generalizing the findings to all ORG chicken meat and in attributing the differences to a specific production factor, given the multiple interrelated factors and practices determining differences between production systems and meat types. Larger datasets are required, encompassing a broad variety of production systems and factors, to disentangle the relative importance of separate factors.

The concentrations of LOP found in the chicken meats and digests indicate potential biological significance. However, it is difficult to determine whether these levels are in a hormetic or pathophysiological range. This could be clarified through in vivo studies assessing the postprandial plasma levels of oxidation products and their potential impact on human health in consumers of chicken meat differing in composition and antioxidant status.

## Figures and Tables

**Table 1:** Characteristics and chemical composition of the finisher phase diets in the conventional and organic production system.

	Production System	
Characteristics	Conventional	Organic
Breed	Ross 308	Sasso (Ruby XL)
Age at slaughter (days)	39–42	73–76
Finishing phase (days)	14	21
Live weight at slaughter (kg)	2.63–2.85	2.30–2.50
Stocking density (kg/m^2^)	42	21
Outdoor access	No	Yes
Indoor enrichments	No	Yes
Diet composition (finisher phase)		
Protein %	21.33	20.56
Fat %	7.40	4.00
Ash %	5.60	5.40
Fiber %	2.60	2.90
Methionine %	0.75	0.30
Lysine %	1.25	0.94
Calcium %	0.73	0.80
Phosphorus %	0.43	0.60
Na %	0.16	0.20
Vitamin A (IU/kg)	13,200	10,000
Vitamin D_3_ (IU/kg)	3630	3000
Vitamin E (mg/kg)	50	55
Se (mg/kg)	0.52	0.30
Iron (mg/kg)	19.80	70
Zn (mg/kg)	66	70
Cu (mg/kg)	19.84	15.40

**Table 2:** Fatty acid profile of heated breast and thigh meats from conventional (CON) and organic (ORG) production system used in the in vitro gastrointestinal digestion of Experiment 1.

Trait	Unit	Breast		Thigh			*p*-Value		
		CON	ORG	CON	ORG	RMSE	S	M	S × M
Total FA	g/100 g	1.06 ± 0.23 ^b^	0.77 ± 0.07 ^b^	2.79 ± 0.80 ^a^	3.05 ± 0.62 ^a^	0.49	0.921	<0.001	0.102
SFA	mg/100 g	343 ± 80.2 ^b^	244 ± 23.8 ^b^	896 ± 262 ^a^	875 ± 188 ^a^	158.5	0.263	<0.001	0.458
MUFA	mg/100 g	316 ± 91.0 ^b^	183± 35.1 ^b^	906 ± 287 ^a^	878 ± 235 ^a^	182.3	0.191	<0.001	0.398
PUFA	mg/100 g	267 ± 51.3 ^c^	235 ± 24.4 ^c^	685 ± 193 ^b^	981 ± 164 ^a^	123.1	0.002	<0.001	<0.001
n-3 PUFA	mg/100 g	22.7 ± 5.63 ^c^	21.8 ± 2.55 ^c^	56.9 ± 16.4 ^b^	85.5 ± 17.1 ^a^	11.60	<0.001	<0.001	<0.001
ALA	mg/100 g	10.7 ± 4.02 ^c^	7.68 ± 1.19 ^c^	41.9 ± 13.7 ^b^	63.4 ± 16.7 ^a^	10.44	0.011	<0.001	0.001
EPA	mg/100 g	2.21 ± 0.87 ^ab^	1.39 ± 0.45 ^b^	2.68 ± 1.27 ^a^	2.28 ± 0.64 ^ab^	0.81	0.031	0.017	0.447
DHA	mg/100 g	2.84 ± 0.88 ^c^	4.94 ± 1.15 ^b^	3.42 ± 1.18 ^c^	7.52 ± 1.39 ^a^	1.10	<0.001	<0.001	0.010
n-6 PUFA	mg/100 g	245 ± 49.8 ^c^	214 ± 22.36 ^c^	627 ± 184 ^b^	894 ± 149 ^a^	116	0.004	<0.001	<0.001
LA	mg/100 g	197 ± 46.7 ^c^	157 ± 18.9 ^c^	565 ± 177 ^b^	803 ± 145 ^a^	111	0.011	<0.001	<0.001
AA	mg/100 g	33.2 ± 3.70 ^c^	45.3 ± 4.20 ^b^	44.9 ± 9.38 ^b^	71.6 ± 10.1 ^a^	7.08	<0.001	<0.001	0.004
n-6/n-3 PUFA	ratio	11.4 ± 3.79	9.8 ± 0.69	11.5 ± 3.20	10.5 ± 0.71	2.40	0.126	0.596	0.741

FA = fatty acid; SFA = saturated fatty acids; MUFA = monounsaturated fatty acids; PUFA = polyunsaturated fatty acids (n-3 PUFA + n-6 PUFA); n-3 PUFA = omega-3 PUFA (C18:3n-3; C20:3n-3; C20:4n-3; C20:5n-3; C22:5n-3; C22:6n-3); ALA = α-linolenic acid (C18:3n-3); EPA = eicosapentaenoic acid (C20:5n-3); DHA = docosahexaenoic acid (C22:6n-3); n-6 PUFA = omega-6 PUFA (C18:2n-6; C18:3n-6; C20:2n-6; C20:3n-6; C20:4n-6; C22:4n-6; C22:5n-6); LA = linoleic acid (C18:2n-6); AA = arachidonic acid (C20:4n-6); n-6/n-3 PUFA = ratio of n-6 PUFA to n-3 PUFA. Values are means ± standard deviation (*n* = 10). Means with different superscripts are significantly different. *p*-values of the main effects of production system (S), meat type (M), and their interaction (S × M) were derived from an ANOVA model. RMSE = root mean square error.

**Table 3:** Fatty acid profile of heated Normal Breast and Wooden Breast meats used in the in vitro gastrointestinal digestion of Experiment 2.

Trait	Unit	Normal Breast	Wooden Breast	RMSE	*p*-Value
Total FA	g/100 g	1.06 ± 0.23	1.42 ± 0.70	0.49	0.156
SFA	mg/100 g	343 ± 80.2	476 ± 244	173	0.131
MUFA	mg/100 g	316 ± 91.0	430 ± 233	167	0.173
PUFA	mg/100 g	267 ± 51.3	353 ± 163	115	0.140
n-3 PUFA	mg/100 g	22.7 ± 5.63	32.9 ± 17.1	12.3	0.090
ALA	mg/100 g	10.7 ± 4.02	21.7 ± 14.8	10.2	0.043
EPA	mg/100 g	2.21 ± 0.87	2.15 ± 0.81	0.79	0.878
DHA	mg/100 g	2.84 ± 0.88	2.63 ± 0.78	0.78	0.592
n-6 PUFA	mg/100 g	245 ± 49.8	320 ± 146	103	0.149
LA	mg/100 g	197 ± 46.7	277 ± 138	96.9	0.110
AA	mg/100 g	33.2 ± 3.70	29.5 ± 8.40	6.15	0.247
n-6/n-3 PUFA	ratio	11.4 ± 3.79	10.2 ± 2.18	2.93	0.389

FA = fatty acid; SFA = saturated fatty acids; MUFA = monounsaturated fatty acids; PUFA = polyunsaturated fatty acids (n-3 PUFA + n-6 PUFA); n-3 PUFA = omega-3 PUFA (C18:3n-3; C20:3n-3; C20:4n-3; C20:5n-3; C22:5n-3; C22:6n-3); ALA = α-linolenic acid (C18:3n-3); EPA = eicosapentaenoic acid (C20:5n-3); DHA = docosahexaenoic acid (C22:6n-3); n-6 PUFA = omega-6 PUFA (C18:2n-6; C18:3n-6; C20:2n-6; C20:3n-6; C20:4n-6; C22:4n-6; C22:5n-6); LA = linoleic acid (C18:2n-6); AA = arachidonic acid (C20:4n-6); n-6/n-3 PUFA = ratio of n-6 PUFA to n-3 PUFA. Values are means ± standard deviation (*n* = 10). *p*-values were determined using *t*-test.

## Data Availability

The original contributions presented in the study are included in the article/Supplementary Material. Further information can be obtained from the corresponding author.

## Supplementary Materials

The following supporting information can be downloaded at https://www.mdpi.com/article/10.3390/foods14193375/s1: Table S1: Composition of the simulated digestive juices (per liter) used in the in vitro digestion model. Figure S1: Visualization of Pearson correlations between variables of oxidation products [protein carbonyl compounds (PCC), thiobarbituric acid reactive substances (TBARS), propanal (PROP), hexanal (HEX), 4-hydroxy-2-nonenal (4-HNE)] in both meats (M) and digests (D) and meat characteristics [n-6 PUFA, n-3 PUFA, total FA, heme-Fe, Anserine, Carnosine, and α-tocopherol] from experiment 1, presented as a heatmap plot. Table S2: Selected oxidation products, their structures, and references.

## Abbreviations

The following abbreviations are used in this manuscript:

4-HNE	4-hydroxy-2-nonenal
CON	conventional production system
DNPH	2,4-dinitrophenylhydrazine
FA	fatty acid
HCD	histidine-containing dipeptides
HEX	hexanal
LDL	low-density lipoproteins
MDA	malondialdehyde
MUFA	monounsaturated fatty acids
ORG	organic production system
PCC	protein carbonyl compounds
PROP	propanal
PUFA	polyunsaturated fatty acids
ROS	reactive oxygen species
SFA	saturated fatty acids
TBA	thiobarbituric acid
TBARS	thiobarbituric acid reactive substances
