# Supplementary material for: Susceptibility of Conventional and Organic Chicken Breast and Thigh Meat to Lipid and Protein Oxidation During Heating and In Vitro Digestion
Source: Foods. 2025 Sep 29;14(19):3375. doi: 10.3390/foods14193375 (PMC12523815; doi:10.3390/foods14193375)
Supplement: Supplementary file 1 [file foods-14-03375-s001.zip › foods-3885499-supplementary.pdf]

Table S1. Composition of the simulated digestive juices (per liter) used in the in vitro digestion model.

	Mouth			Stomach			Duodenum					
	Saliva (pH 6.8)			Gastric juice (pH 1.3)			Duodenal juice (pH 8.1)			Bile (pH 8.2)		
A												
	KCI	0.900	g/l	KCI	0.820	g/l	KCI	0.560	g/l	KCI	0.380	g/l
	NaCI	0.300	g/l	NaCI	2.750	g/l	NaCI	7.010	g/l	NaCI	5.260	g/l
	Na_2SO_4	0.570	g/l	$CaCI_2$ $2H_2O$	0.400	g/l	$MgCI_2$	0.050	g/l	$NaHCO_3$	5.790	g/l
	NaH_2PO_4	0.900	g/l	NaH_2PO_4	0.270	g/l	KH_2PO_4	0.080	g/l	HCI 37%	0.150	ml/l
	$NaHCO_3$	1.690	g/l	NH_4CI	0.037	g/l	$NaHCO_3$	3.390	g/l	Urea	10.000	ml/l
	KSCN	0.200	g/l	HCI 37%	7.500	ml/l	HCI 37%	0.180	ml/l	BSA	1.800	g/l
B												
	Urea	8.000	ml/l	Urea	6.000	ml/l	Urea	4.000	ml/l	Bile	30.000	g/l
	Uric acid	0.012	g/l	BSA	1.000	g/l	BSA	1.000	g/l	$CaCI_2$ $2H_2O$	0.222	g/l
	Mucin	0.025	g/l	Mucin	3.000	g/l	Pancreatin	9.000	g/l			
	Amylase	0.290	g/l	Pepsin	2.500	g/l	Lipase	1.500	g/l			
	$NaNO_2$	0.007	g/l	Glucose	0.650	g/l	$CaCI_2.2H_2O$	0.200	g/l			
				Glucosamine-HCI	0.330	g/l						
				Ascorbic acid	0.018	g/l						
				Glucuronic acid	0.020	g/l						
				$FeSO_4$ $7H_2O$	0.011	g/l						
C												
				H_2O_2 (30%)	20.000	µl/l						

Solution A was prepared in advance and stored at 4°C, whereas solution B was freshly prepared immediately before mixing with solution A. Chemical C was added to complete the digestive juice (Van Hecke et al., 2018).

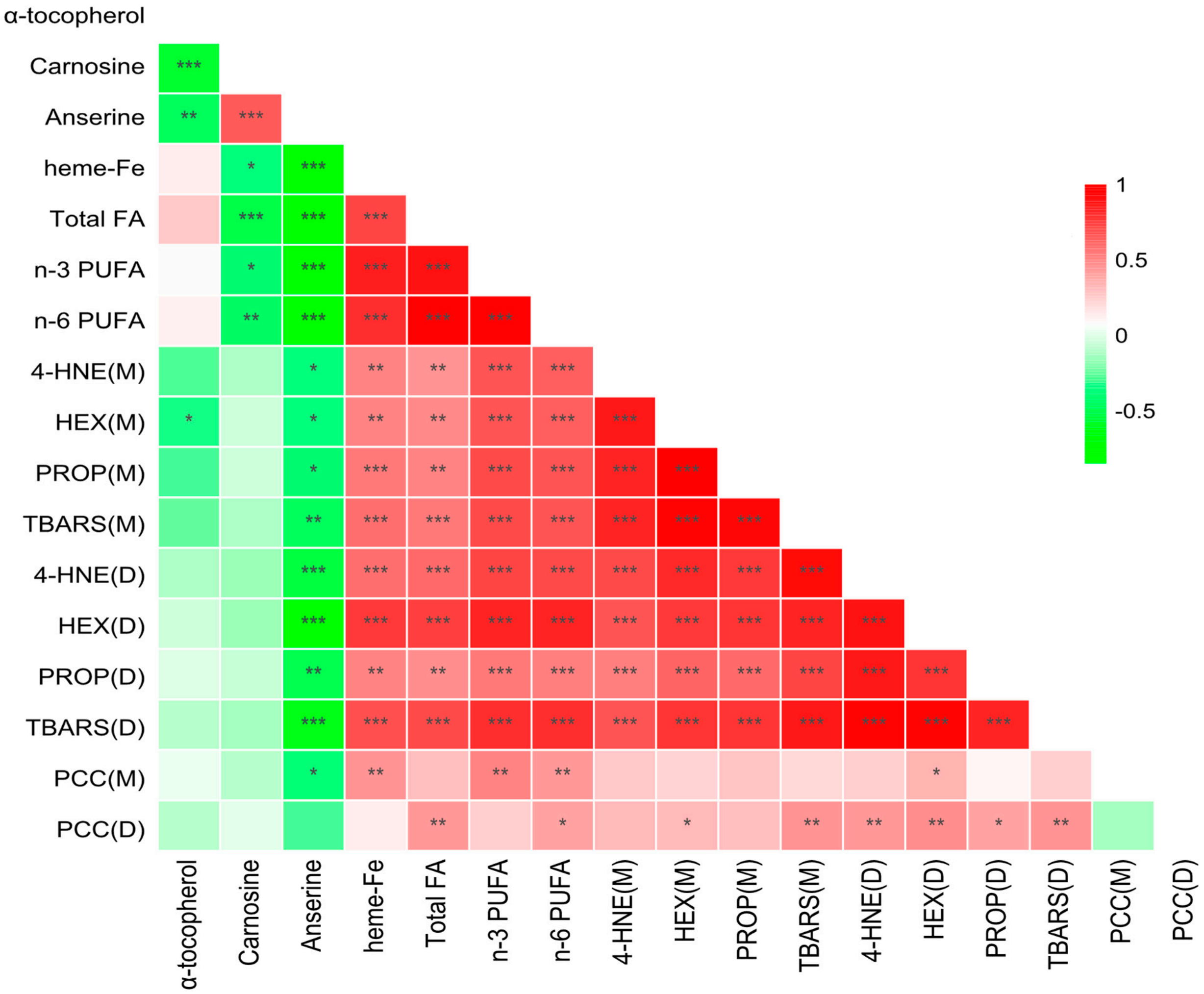

Figure S1. Visualization of the Pearson correlation coefficients between the variables of oxidation products [protein carbonyl compounds, PCCs; thiobarbituric acid reactive substances, TBARS; propanal, PROP; hexanal, HEX; 4-hydroxy-2-nonenal, 4-HNE) in both meats (M) and digests (D)] and meat characteristics [n-6 PUFA, n-3 PUFA, Total FA, heme-Fe, Anserine, Carnosine and α-tocopherol] in Experiment 1, presented via a heatmap plot. The color gradient from red to green indicates the Pearson correlation coefficients from 1 to -1: red for strong positive correlations and green for strong negative correlations. * $P \leq 0.05$, ** $P \leq 0.01$, *** $P < 0.001$.

Table S2. Selected oxidation products, their structures, and references

name	structure	reference
Malondialdehyde (MDA)	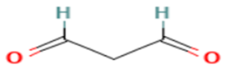	https://pubchem.ncbi.nlm.nih.gov/compound/10964
4-hydroxy-2-nonenal (4-HNE)	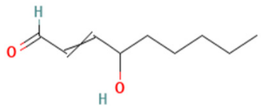	https://pubchem.ncbi.nlm.nih.gov/compound/1693
Hexanal (HEX)	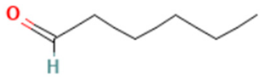	https://pubchem.ncbi.nlm.nih.gov/compound/6184
Propanal (PROP)	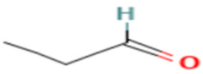	https://pubchem.ncbi.nlm.nih.gov/substance/447277250
